# Supplementary material for: Characteristics of Chimeric West Nile Virus Based on the Japanese Encephalitis Virus SA14-14-2 Backbone
Source: Viruses. 2021 Jun 29;13(7):1262. doi: 10.3390/v13071262 (PMC8309971; doi:10.3390/v13071262)
Supplement: Supplementary file 1 [file viruses-13-01262-s001.zip › viruses-1173280-supplementary materials.pdf]

Supplementary Materials:

1. K, L, M, KL and KLM Amplification

Fragment K, L and M were amplified by primers in Table 1 according to PCR reaction system. And the JEV SA14-14-2 cDNA were used as templates for fragment K (Table S1) and M (Table S3) amplification. WNV prME DNA were used as templates for fragment L (Table S2) amplification.

Table S1. Fragment K PCR reaction system.

Component	Amount, μL
ddH$_2$O	30
5×Phusion HP Reaction Buffer	10
Trans 2.5 mM dNTPs	4
Primer-F (K-F, 10μM)	2.5
Primer-R (K-R, 10μM)	2.5
Templates (JEV SA14-14-2 cDNA)	0.5
NEB Phusion HF DNA Polymerase	0.5
Total volume	50 μL

Cycling conditions: 98 °C 30 s; (98 °C 7 s, 57 °C 30 s, 72 °C 30 s; 35 cycles); 72 °C 10 min, 4 °C ∞.

Table S2. Fragment L PCR reaction system.

Component	Amount, μL
ddH$_2$O	30
5×Phusion HP Reaction Buffer	10
Trans 2.5 mM dNTPs	4
Primer-F (L-F, 10μM)	2.5
Primer-R (L-R, 10μM)	2.5
Templates (WNV prME)	0.5
NEB Phusion HF DNA Polymerase	0.5
Total volume	50 μL

Cycling conditions: 98 °C 30 s; (98 °C 7 s, 55 °C 30 s, 72 °C 1 min; 5 cycles); (98 °C 7 s, 60 °C 30 s, 72 °C 1 min; 30 cycles); 72 °C 10 min, 4 °C ∞.

Table S3. Fragment M PCR reaction system.

Component	Amount, μL
ddH$_2$O	30
5×Phusion HP Reaction Buffer	10
Trans 2.5 mM dNTPs	4
Primer-F (M-F, 10μM)	2.5
Primer-R (M-R, 10μM)	2.5
Templates (JEV SA14-14-2 cDNA)	0.5
NEB Phusion HF DNA Polymerase	0.5
Total volume	50 μL

Cycling conditions: 98 °C 30 s; (98 °C 7 s, 55 °C 30 s, 72 °C 30 s; 5 cycles); (98 °C 7 s, 60 °C 30 s, 72 °C 30 s; 30 cycles); 72 °C 10 min, 4 °C ∞.

The amplified DNA (K, L and M) were evaluated by 1% agarose gel electrophoresis and subsequent ethidium bromide staining, and purified by Gel Extraction Kit (Omega Bio-tek, Inc., Norcross, GA, USA). Followed, the fragment KL (Table S4) and KLM (Table S5) were amplified by the following overlap PCR reaction system.

Table S4. Fragment KL PCR reaction system.

Component	Amount, μL
ddH_2O	29.3
5×Phusion HP Reaction Buffer	10
Trans 2.5 mM dNTPs	4
Primer-F (K-F, 10μM)	2.5
Primer-R (L-R, 10μM)	2.5
Templates (Fragment K)	0.2
Templates (Fragment L)	1
NEB Phusion HF DNA Polymerase	0.5
Total volume	50 μL

Cycling conditions: 98 °C 30 s; (98 °C 7 s, 55 °C 30 s, 72 °C 1 min 15 s; 5 cycles); (98 °C 7 s, 57 °C 30 s, 72 °C 1 min 15 s; 30 cycles); 72 °C 10 min, 4 °C ∞.

Keeping the equal moles of K and L will help KL production. That is *n*(K):*n*(L) = 1:1.

Table S5. Fragment KLM PCR reaction system.

Component	Amount, μL
ddH_2O	29.3
5×Phusion HP Reaction Buffer	10
Trans 2.5 mM dNTPs	4
Primer-F (K-F, 10μM)	2.5
Primer-R (M-R, 10μM)	2.5
Templates (Fragment KL)	1
Templates (Fragment M)	0.2
NEB Phusion HF DNA Polymerase	0.5
Total volume	50 μL

Cycling conditions: 98 °C 30 s; (98 °C 7 s, 60 °C 30 s, 72 °C 1 min 45 s; 35 cycles); 72 °C 10 min, 4 °C ∞.

Keeping the equal moles of KL and M will help KLM production. That is *n*(KL):*n*(M) = 1:1.

The fragment KLM amplified were evaluated by 1% agarose gel electrophoresis and subsequent ethidium bromide staining, and purified by Gel Extraction Kit (Omega Bio-tek, Inc., Norcross, GA, USA).

2. Construction of pACYC177-Linker

Linker with multiple cloning sites (*Bam*H I-*Not* I-*Bsr*G I-*Kpn*2 I-*Bsi*W I-*Xba* I-*Psi* I) (Table S6a) was introduced into the pACYC177 at *Bam*H I (3320 bp) and *Psi* I (3483 bp) restriction sites for pACYC177-linker construction. Two primers Linker- F′/R′ was synthesized by Comate Bioscience Co., Ltd. (Changchun, Jilin, China) (Table S6c). The equal moles of two primers were mixed and incubated at 95 °C for 5 min, and the temperature of the mixture was lowered gradually to 65 °C lasting for 10 min, and then the sample temperature was decreased to room temperature [27]. The mixture of Linker-F′/R′ could form a double-stranded fragment with a sticky (*Bam*H I) and a blunt (*Psi* I) ends (Table 6b) by the procedure. The fragment was used directly to ligate with digested pACYC177 (Table S7) to construct pACYC177-linker (Table S8).

Do select sequences in Table S6c to synthesize primers! Linker-R′ in Table S6b is easily to be as 5′-3′ direction to be synthesized, which will result in the SAME DIRECTION primers and lead to the failure of experiment!

Table S6. Linker sequences.

Linker names		Linker sequences with multiple cloning sites<br>*Bam*H I-*Not* I-*BsrG* I-*Kpn*2 I-*BsiW* I-*Xba* I-*Psi* I
a	Linker-F	5' GGATCCATCGCGGCCGCATCTGTACAATCTCCGGAATCCGTACGATCTCTAGACACTTATAA 3'
	Linker-R	3' CCTAGGTAGCGCCGGCGTAGACATGTTAGAGGCCTTAGGCATGCTAGAGATCTGTGAATATT 5'
b	Linker-F'	5' GATCCATCGCGGCCGCATCTGTACAATCTCCGGAATCCGTACGATCTCTAGACACTTA 3'
	Linker-R'	3' GTAGCGCCGGCGTAGACATGTTAGAGGCCTTAGGCATGCTAGAGATCTGTGAAT 5'
c	Linker-F'	5' GATCCATCGCGGCCGCATCTGTACAATCTCCGGAATCCGTACGATCTCTAGACACTTA 3'
	Linker-R'	5' TAAGTGTCTAGAGATCGTACGGATTCCGGAGATTGTACAGATGCGGCCGCGATG 3'

Restriction endonuclease sites GGATCC (*Bam*H I), GCGGCCGC (*Not* I), TGTACA (*Bsr*G I), TCCGGA (*Kpn*2 I), CGTACG (*Bsi*W I), TCTAGA (*Xba* I), and TTATAA (*Psi* I) are underlined. a, linker sequence with uncut restriction sites; b, linker sequence with sticky (*Bam*H I) and blunt (*Psi* I) ends; c, 5'-3' direction of Linker-F'/R'. F indicates forward primer, and R indicates reverse primer.

Table S7. Protocol for pACYC177 digestion.

Component	Amount, μL
ddH$_2$O	Variable
10×FastDigest Green Buffer	5
pACYC177	2.5 μg
FastDigest *Bam*H I	2.5
FastDigest *Psi* I	2.5
Total volume	50 μL

The mixtures were incubated at 37 °C for 1 h.

The digested pACYC177 plasmids were evaluated by 1% agarose gel electrophoresis and subsequent ethidium bromide staining, and purified by Gel Extraction Kit (Omega Bio-tek, Inc., Norcross, GA, USA) for ligation (Table S8).

Table S8. Ligation protocol.

Component	Amount, μL
T4 DNA Ligase Buffer (10×)	1
Vector DNA (digested pACYC177)	20 ng
Inserted DNA (Linker)	Variable
T4 DNA Ligase	1
ddH$_2$O	Variable
Total volume	10 μL

n (Vector DNA): *n* (Inserted DNA) =1:3~10.

The ligation product was incubated at 25 °C for 1 h.

5 μL of ligation product was transformed into *E. coli* DH5α competent cells (Takara, Dalian, Liaoning, China), recovering cells, plating and culturing at 37 °C for pACYC177-linker production.

3. Construction of pACYC177-KLM

The purified KLM and pACYC177-linker were digested by *Not* I and *Kpn*2 I respectively (Table S9, S10). The digested products were purified by AxyPrep PCR Clean-up Kit (Corning, Glendale, Arizona, USA). The purified digested KLM was ligated onto pACYC177-linker by T4 DNA Ligase (New England Biolabs, Ipswich, MA, USA) (Table S11).

Table S9. Protocol for Fragment KLM digestion.

Component	Amount, μL
ddH_2O	Variable
10×FastDigest Green Buffer	5
PCR product (KLM)	2.5 μg
FastDigest *Not* I	2.5
FastDigest *Kpn*2 I	2.5
Total volume	50 μL

Table S10. Protocol for pACYC177-linker digestion.

Component	Amount, μL
ddH_2O	Variable
10×FastDigest Green Buffer	5
pACYC177-linker	2.5 μg
FastDigest *Not* I	2.5
FastDigest *Kpn*2 I	2.5
Total volume	50 μL

The mixtures were incubated at 37 °C for 1 h.

The restriction digested fragments (KLM and pACYC177-linker) were purified by AxyPrep PCR Clean-up Kit (Corning, Glendale, Arizona, USA) for ligation (Table S11).

Table S11. Ligation protocol.

Component	Amount, μL
T4 DNA Ligase Buffer (10×)	1
Vector DNA (digested pACYC177-linker)	20 ng
Inserted DNA (digested KLM)	Variable
T4 DNA Ligase	1
ddH_2O	Variable
Total volume	10 μL

n (Vector DNA): n (Inserted DNA) =1:3~10.

The ligation product was incubated at 25 °C for 1 h.

5 μL of ligation product was transformed into *E. coli* HB101 competent cells (Takara, Dalian, Liaoning, China), recovering cells, plating and culturing at 37 °C for pACYC177-KLM production.

4. Construction of pACYC177-KLM-F345-HDVr-T7ter (pChiVax-WN01)

The pACYC177-KLM (Table S12) and the pFLJEV (Table S13) were digested by *Kpn*2 I and *Psi* I respectively. The digested products were purified by AxyPrep PCR Clean-up Kit (Corning, Glendale, Arizona, USA) or evaluated by 1% agarose gel electrophoresis and subsequent ethidium bromide staining, and purified by Gel Extraction Kit. The purified F345-HDVr-T7ter was ligated onto pACYC177-KLM to construct full-length infectious clone pACYC177-KLM-F345-HDVr-T7ter (pChiVax-WN01) (Table S14).

Table S12. Protocol for pACYC177-KLM digestion.

Component	Amount, μL
ddH_2O	Variable
10×FastDigest Green Buffer	5
pACYC177-KLM	2.5 μg
FastDigest *Kpn*2 I	2.5
FastDigest *Psi* I	2.5
Total volume	50 μL

The mixture was incubated at 25 °C for 1 h.

Table S13. Protocol for pFLJEV digestion.

Component	Amount, μL
ddH_2O	Variable
10×FastDigest Green Buffer	5
pFLJEV	2.5 μg
FastDigest *Kpn*2 I	2.5
FastDigest *Psi* I	2.5
Total volume	50 μL

The mixture was incubated at 25 °C for 1 h.

The restriction digested fragments pACYC177-KLM and F345-HDVr-T7ter purified were used for ligation (Table S14).

Table S14. Ligation protocol.

Component	Amount, μL
T4 DNA Ligase Buffer (10×)	1
Vector DNA (digested pACYC177-KLM)	20 ng
Inserted DNA (digested F345-HDVr-T7ter)	Variable
T4 DNA Ligase	1
ddH_2O	Variable
Total volume	10 μL

n (Vector DNA): *n* (Inserted DNA) =1:3~10.

The mixture was incubated at 25 °C for 1 h.

5 μL of ligation product was transformed into *E. coli* HB101 competent cells (Takara, Dalian, Liaoning, China), recovering cells, plating and culturing at room temperature for pACYC177-KLM-F345-HDVr-T7ter (pChiVax-WN01) production.

Table S15. Neuroinvasiveness of ChiVax-WN01 and JEV SA14-14-2 in Mice. Groups (n = 8~10) of 4-week-old female BALB/c mice were inoculated i.p. with 10^5 PFU~10^1 PFU of ChiVax-WN01 and JEV SA14-14-2 respectively. The mice were monitored for 15 days.

Virus (Strain)	Dose (PFU)	Survival (no. Survived/Total)
JEV SA14-14-2	10^5	10/10
	10^5	10/10
	10^4	8/8
ChiVax-WN01	10^3	10/10
	10^2	10/10
	10^1	10/10
PBS	100 μL	10/10

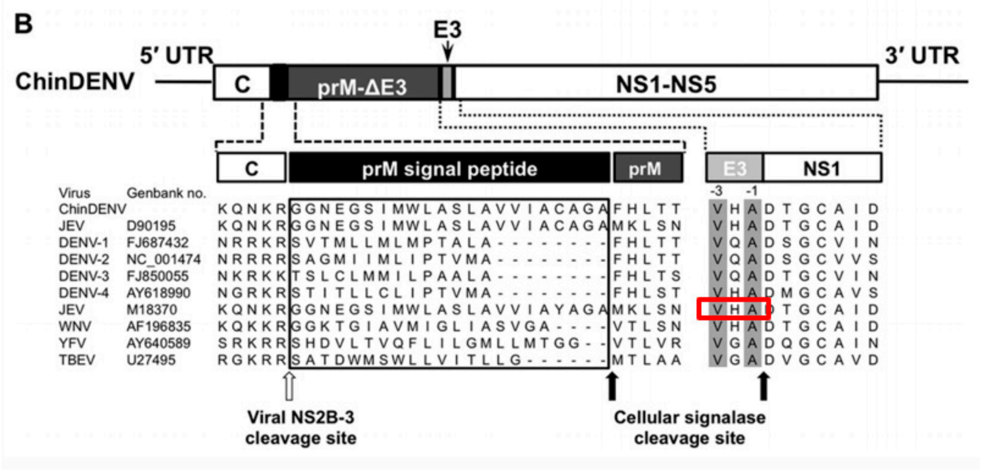

Figure S1. Alignment of flavivirus amino acid sequences around the C-prM and E-NS1 junctions [21]. The last three amino acids "VHA" in the JEV E protein were listed in the red rectangle.

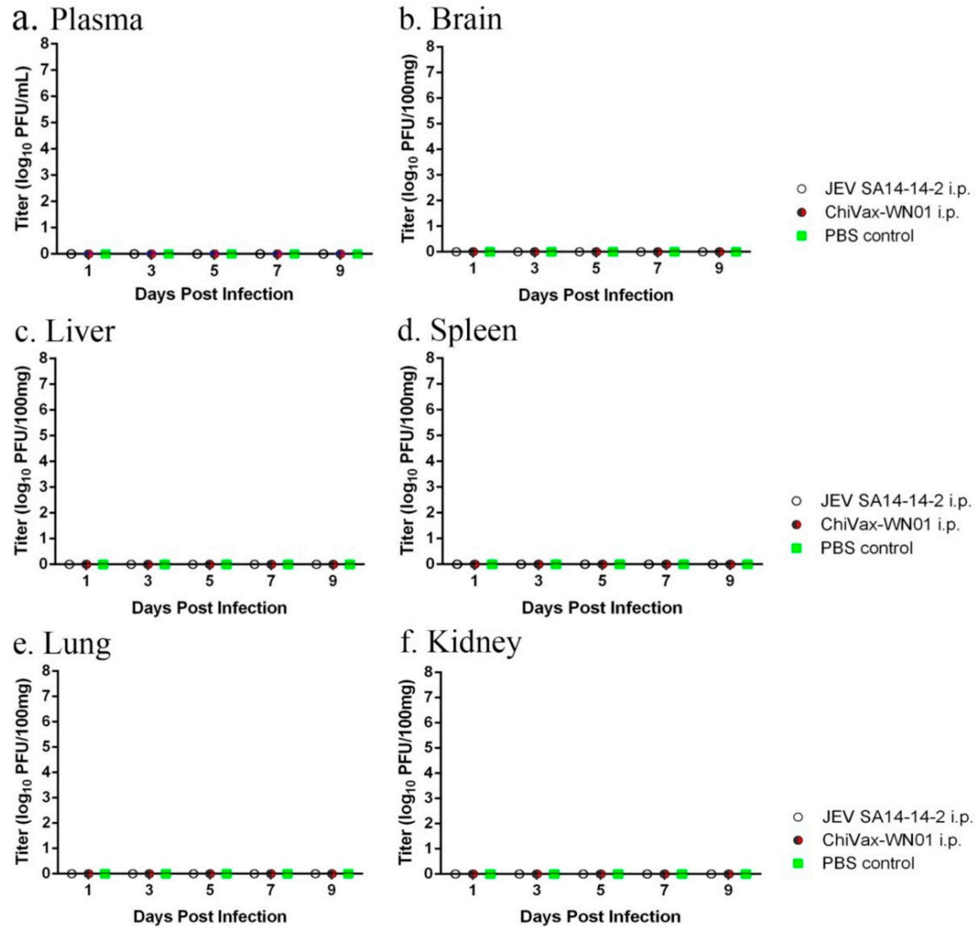

Figure S2. Organ titers following i.p. inoculation. 4-week-old female BALB/c mice were inoculated i.p. with 100 PFU of JEV SA14-14-2 or ChiVax-WN01, respectively. Three mice in each group were euthanized on day 1, 3, 5, 7 and 9, and plasma and organs (brain, liver, spleen, lung and kidney) were collected for titration by plaque assay.
